# Are All Oral COX-2 Selective Inhibitors the Same? A Consideration of Celecoxib, Etoricoxib, and Diclofenac

**DOI:** 10.1155/2018/1302835

**Published:** 2018-12-09

**Authors:** Chris Walker

**Affiliations:** Pfizer, Walton Oaks, UK

## Abstract

Nonsteroidal anti-inflammatory drugs (NSAIDs) have been widely used for the treatment of arthritic conditions. Drugs in this heterogeneous class alleviate pain and inflammation by inhibiting cyclooxygenase-2 (COX-2). Cyclooxygenase-1 (COX-1) inhibition has traditionally been associated with increased gastrointestinal (GI) harm, whereas increased COX-2 selectivity has more recently become associated with greater risk of cardiovascular (CV) harm. When the entirety of data is considered, NSAIDs can be seen to exhibit a range of COX isoform selectivity, with all oral NSAIDs appearing to be associated with an increase in CV events. This review focuses on a comparison of the efficacy and the GI and CV safety profiles of three commonly used NSAIDs—celecoxib, etoricoxib, and diclofenac—using direct comparisons where available. While all three treatments are shown to have comparable efficacy, there are differences in their safety profiles. Both celecoxib and etoricoxib are associated with less GI harm than diclofenac despite the similarity of its COX-2 selectivity to celecoxib. Each of the three medicines under consideration is associated with a similar overall risk of CV events (fatal and nonfatal heart attacks and strokes). However, there are consistent differences in effects on blood pressure (BP), reported both from trials using ambulatory techniques and from meta-analyses of randomized trials, reporting investigator determined effects, with etoricoxib being associated with a greater propensity to destabilize BP control than either diclofenac or celecoxib.

## 1. Introduction

Nonsteroidal anti-inflammatory drugs (NSAIDs) have been widely prescribed for decades to reduce pain and inflammation in patients with chronic arthritic conditions. NSAIDs provide symptomatic relief by inhibiting cyclooxygenase (COX) enzymes, with a subsequent reduction in the prostaglandin mediators of pain. Beneficial effects were believed to be predominantly mediated through inhibition of the largely inducible form of the enzyme, known as cyclooxygenase-2 (COX-2), whereas housekeeping functions, such as gastric homeostasis, were believed to be largely mediated through cyclooxygenase-1 (COX-1) [1, 2].

While all NSAIDs must inhibit COX-2 to have any effect on pain/inflammatory processes, NSAIDs are a chemically diverse group of medicines with significant heterogeneity in chemical structure and properties [3, 4]. They show substantial variation in selectivity for COX-2 over COX-1, with this variable being best considered as continuous rather than simply dichotomous, where medicines are presented as either COX-2 selective or nonselective [5].

Medicines with proprietary names ending in “coxib”, such as celecoxib or etoricoxib, are traditionally thought of as the most selective in their effect on the COX-2 enzymes and were developed to reduce gastrointestinal (GI) toxicity associated with the use of traditional NSAIDs, but they have been characterized by some as having less favorable cardiovascular (CV) profiles than agents that are nonselective [5]. The term coxib, however, has no pharmacological meaning, and different in vitro assay systems, measuring selectivity ratios for COX-1 and COX-2 enzymes, can produce markedly different results [6, 7]. Most typically, celecoxib is seen to possess a similar COX-2 selectivity ratio to diclofenac (IC_50_ ratios of 30 and 29, respectively, is commonly cited) [7, 8] with etoricoxib typically producing a ratio an order of magnitude higher in vitro [9]. In contrast, in vivo findings display marked differences in an effect on COX-1, when comparing therapeutic concentrations of celecoxib and diclofenac, suggesting that simple investigation of in vitro assays may be of limited use [10].

Results of a large meta-analysis of randomized controlled trials have shown that diclofenac was associated with a risk of CV harm comparable to coxibs when considered as a broad class [11]. As a consequence, prescribing guidance generated by the European Medicines Agency [12] and guidelines from the European Society of Cardiology [5] has been updated to include the same contraindications (in patients with established CV disease, peripheral arterial disease, and/or cerebrovascular disease) for prescription-strength diclofenac (150 mg/day) as those included for the agents that have always been considered COX-2 selective inhibitors. In addition, as of January 2015, oral diclofenac is no longer available as an over-the-counter medicine in the UK [13].

This review compares the available head-to-head and placebo-controlled data on the efficacy and the GI and CV safety of celecoxib, etoricoxib, and diclofenac, with the aspiration of highlighting the similarities and differences between agents.

## 2. Celecoxib, Etoricoxib, and Diclofenac Have Comparable Efficacy in Patients With Arthritic Conditions 

Evidence considering the efficacy of the three medicines demonstrates that they are essentially similar in terms of the level of pain relief that they provide for patients with chronic arthritic conditions. The evidence is particularly compelling for osteoarthritis (OA).  Table 1 summarizes three 12-week studies, in which etoricoxib (30 mg once daily [OD]) was shown to be noninferior to celecoxib (200 mg OD), with both etoricoxib and celecoxib being significantly more effective than placebo (*p* < 0.001 for both) as assessed by commonly used pain scales [14, 15].

Similarly, 6-week trials have shown comparable levels of efficacy when diclofenac (50 mg three times daily [TID]) was compared with celecoxib (100 mg twice daily [BID]) or etoricoxib (60 mg OD or 30–90 mg OD) for the treatment of OA of the knee [16, 17]. Etoricoxib (30–90 mg OD) was also comparable to diclofenac over a longer, 14-week time frame in patients with OA of the knee [18]. In contrast, a 12-week study in patients with severe OA of the hip awaiting hip surgery failed to demonstrate noninferiority of celecoxib (200 mg OD) to diclofenac (50 mg TID), with greater pain relief observed in the diclofenac arm of this severe pain model [19].

In other main arthritic conditions, specifically rheumatoid arthritis (RA) and ankylosing spondylitis (AS), there are no direct head-to-head trials comparing celecoxib and etoricoxib. In patients with adult-onset RA, the efficacy of celecoxib (200 mg BID) was shown to be similar to that of diclofenac (75 mg BID) in a 24-week study [20]. In a number of noninferiority 12-week studies in patients with AS, celecoxib (200 mg OD or 200 mg BID) had similar efficacy to diclofenac (75 mg OD or BID, or 50 mg TID) [21–23]. The efficacy of etoricoxib (90–120 mg OD) has been well studied in RA [24] and AS [25], with naproxen (1000 mg/day) being the comparator of choice.

The head-to-head studies described above largely demonstrate the comparable efficacy of celecoxib, etoricoxib, and diclofenac in alleviating pain and improving function in patients with OA, RA, and AS.

In addition to its anti-inflammatory and analgesic properties, celecoxib, but not diclofenac, was shown to have potentially disease-modifying properties in patients with AS [26–28]. This putative property does not appear to have been studied for etoricoxib at the time of writing. Suggestions of disease-modifying properties have also been tentatively put forward for celecoxib in OA, perhaps relating to COX-independent pathways that effect many processes including chondrocyte apoptosis [29]. Similar pathways are believed to play an important role in cancer models, where again a role for celecoxib in COX-independent apoptotic pathways is commonly reported [30], with more recent research suggesting that there is at least one gene that modulates the effects of NSAIDs in a prostaglandin independent manner [31]. While many of these findings have yet to translate into clinical benefits directly, the efficacy and safety of the three agents described should not be considered solely on the basis of their effects on the two COX isoforms.

## 3. Comparison of Safety Profiles of Celecoxib, Etoricoxib, and Diclofenac 

While meta-analyses and randomized controlled trials have evaluated the safety of different NSAIDs using direct and indirect methods [11], few randomized controlled trials have directly compared safety outcomes between celecoxib and etoricoxib. Each, however, has been directly compared with diclofenac. The following section considers upper GI safety (gastropathy), before moving on to the less well studied topic of lower GI safety (enteropathy).

### 3.1. Upper Gastrointestinal Safety (Gastropathy)

NSAID-induced decreases in mucosal levels of prostaglandins (driven by inhibition of COX-1) correlate with both gastric and small-bowel damage [32]. Proposed mechanisms of damage to the stomach involve prostaglandin-mediated increase in gastric acid secretion, decrease in mucus and bicarbonate secretion, decrease in cell proliferation, and decrease in mucosal blood flow [33]. In addition, topical effects of the NSAIDs on the mucosal lining, that are not dependent on COX inhibition, are believed to play a significant role, including the ability of NSAIDs to uncouple oxidative phosphorylation with a resultant decrease in ATP production and cellular integrity [32].

The largest independent meta-analysis of individual patient-level data [11] could at first appraisal be taken to suggest that there are no differences between diclofenac and coxibs, both being associated with a similar elevation in the risk of ulcer complications (perforations, obstructions, and bleeds) when compared with placebo (rate ratio [95% CI], coxibs 1.81 [1.17–2.81]; diclofenac 1.89 [1.16–3.09]). However, a more detailed evaluation of individual medicines and symptomatic upper GI events (symptomatic ulcers plus perforations, obstructions, and bleeds) demonstrated that both of the currently available coxibs, considered at any dose, were associated with a significant reduction in the risk of harm when compared with any dose of diclofenac (rate ratio [95% CI], celecoxib versus diclofenac 0.54 [0.37–0.79],* p* = 0.0006; etoricoxib versus diclofenac 0.70 [0.57–0.85],* p* = 0.0002) [11].

Several of the larger individual clinical trials considered in the Bhala meta-analysis [11] have explored the GI safety profile of celecoxib or etoricoxib in comparison with diclofenac. An initial trial of celecoxib (CLASS) [34] only considered upper GI endpoints with supratherapeutic doses (800 mg/day) of celecoxib compared to pooled data for the comparator NSAIDs (diclofenac [75 mg BID] and ibuprofen [800 mg TID]). It demonstrated a significant difference in uncomplicated upper GI events, but not in complicated events [34]; however, no individual analysis was presented comparing celecoxib and diclofenac.

A large trial program comparing etoricoxib and diclofenac (MEDAL) enrolled patients with OA and RA across three trials: EDGE (OA, mean 9 months), EDGE II (RA, mean 19 months), and the MEDAL study (OA and RA, mean 20 months and up to 42.3 months) (Table 2) [35–38]. In the MEDAL program, no difference was observed between etoricoxib and diclofenac in complicated events (complicated bleeding, perforation, or obstruction), but there were significantly fewer uncomplicated events (uncomplicated bleeding or ulcer) with etoricoxib when compared with diclofenac (Table 3) [39]. This large trial program, primarily designed to assess the relative CV safety of the two medicines, can be considered highly pragmatic as it permitted open-label proton pump inhibitor (PPI) use (~39% of patients) and low-dose aspirin use (< 100 mg in ~35% of patients) (Table 2) [39]. A GI benefit of etoricoxib was observed in both of these subgroups for the endpoint of uncomplicated events; however, a subgroup analysis of patients with and without PPI use showed no difference in complicated upper GI events between etoricoxib and diclofenac [39].

Similar benefits for either etoricoxib or celecoxib in relation to GI tolerability are largely seen in other trials [35] or pooled analyses of trials [40, 41], demonstrating a statistically significant reduction in harm associated with the use of COX-2 selective agents when compared to diclofenac, as illustrated in Table 3.

### 3.2. Lower Gastrointestinal Safety (Enteropathy)

In the small bowel the role of prostaglandin production appears to be less important to the pathogenic effects of NSAIDs [32]. The mechanisms postulated are more complex and include a range of effects that are believed to locally irritate cells of the epithelial lining, resulting in an increase in permeability of these cells. The effect on mitochondrial function, described in the gastropathy section, is likely to play a role, along with important contributions to NSAIDs enteropathy from enteric bacteria, bile, and the degree of enterohepatic recycling (EHR) that a particular NSAID undergoes [32, 42].

The potential of different NSAIDs to cause enteropathy has been less well evaluated than NSAID-related gastropathy. The complexity of GI endpoints evaluated in clinical trials of NSAIDs has evolved slightly over time (see Table 2), with more recently conducted studies evaluating the GI safety of NSAIDs below the duodenum by measuring bleeding of defined or presumed occult GI origin (the latter including small-bowel blood loss) [43], as well as documenting the increasingly rare cases of ulcer complications from both the upper and lower GI tract (Table 2) [43].

The CONDOR trial, building on data from a single-center study of similar design [44], demonstrated a significantly lower incidence of clinically significant GI events with celecoxib 200 mg BID compared with diclofenac (75 mg slow release BID) in conjunction with 20 mg OD of omeprazole for gastroprotection (Table 3) [43]. The majority of the observed difference was driven by blinded adjudicated hemoglobin drops of ≥ 2 g/dL of either presumed small-bowel blood loss (10 events for celecoxib versus 53 for diclofenac + PPI) or hemoglobin drops with a defined lesion in the upper GI tract (five events for celecoxib versus 20 for diclofenac + PPI) [43].

The subtle differences in lower GI harm observed in the CONDOR trial [43] were not observed in the MEDAL program, where there was no difference in the rate of lower GI events between etoricoxib and diclofenac (Table 3) [35, 45]. The prohibition of aspirin use in the CONDOR trial may explain at least part of this apparent difference (~35% used low-dose aspirin in the MEDAL program) [35, 45], along with the more rigorous assessment of endpoints and higher frequency of data capture of endpoints in the CONDOR trial [43]. In the latter trial, hemoglobin was assessed at each study visit at months 1, 2, 3, and 6 [43], while in the MEDAL program patients were assessed every 4 months [45].

Mechanistic differences between the drugs, beyond COX-2 selectivity, could also account for subtle differences in their GI safety profiles. In an aged rat model of NSAID-induced lower GI injury, celecoxib, a nonacidic compound, was associated with less intestinal damage than the acidic compounds etoricoxib and diclofenac, with the authors suggesting that acidity was a key determinant of the level of injury observed [46]. Molecular acidity has also been proposed as a determinant of enteropathy by other authors [32, 47, 48].

### 3.3. Cardiovascular Safety

The COX-2 selective inhibition that is responsible for a reduction in GI harm was also linked to early theoretical concerns regarding CV safety [49, 50]. In a placebo-controlled polyp prevention trial, rofecoxib was associated with increased CV risk of thromboembolic outcomes (APPROVe) [51] and was subsequently withdrawn from the market by the manufacturer. Data from celecoxib trials in the same investigational setting was not consistent (APC and PreSAP trials) with that seen with rofecoxib [52, 53]. None of these trials were designed or powered to assess CV outcomes [52, 53], and no similar data exists considering diclofenac in a placebo-controlled polyp prevention setting.

The paucity of well-powered clinical trials prospectively addressing the question of NSAID CV safety has encouraged various authors to conduct meta-analyses of randomized controlled trials [11, 54] and observational data [55] to try to address this question (Table 4). The largest meta-analysis of 754 randomized trials (353,809 patients) suggested that the CV risk of coxibs was similar to that of diclofenac (150 mg/day) [11]. Neither celecoxib nor etoricoxib differed significantly from diclofenac in major vascular events (Table 4) [11]. In another meta-analysis of 31 randomized trials (116,429 patients) assessing CV risk (Antiplatelet Trialists' Collaboration composite outcome) versus placebo, both celecoxib and etoricoxib were also comparable to diclofenac and other NSAIDs [54]—a result supported by the largest meta-analysis of observational data (Table 4) [55, 56]. In most of these meta-analyses, a mixture of direct and indirect comparisons was used to draw the conclusions, as there are no direct data comparing the CV safety of celecoxib and etoricoxib.

When looking at direct comparisons between our three chosen agents, the data are far more limited but tell a largely consistent story. The MEDAL program, introduced in the prior section on GI safety, was primarily designed as a noninferiority trial to address the relative CV safety of etoricoxib compared with diclofenac through the accumulation of over 640 CV thrombotic endpoints [35]. During the conduct of the MEDAL program, the dose of etoricoxib was reduced from 90 mg/day to 60 mg/day in accordance with a downward change to the recommended dose for patients with RA [57, 58]. The rate of thrombotic CV events was comparable in patients treated with etoricoxib or diclofenac in the MEDAL program (hazard ratio [95% CI], 0.95 [0.81–1.11]) (Table 4) [35]. The only direct comparison considering more than a dozen endpoints from trial data published for celecoxib (800 mg/day) and diclofenac (150 mg/day) is taken from a post hoc analysis and demonstrated a similar incidence of CV thrombotic events for celecoxib and diclofenac based on the 80 endpoints captured (Table 4) [59].

More recently, a further large long-term trial [60] investigating the CV safety of celecoxib, compared with the traditional NSAIDs ibuprofen and naproxen in patients with elevated baseline CV risk prospectively demonstrated that celecoxib (mean daily dose 209 mg) was noninferior to ibuprofen (mean daily dose 2045 mg) and naproxen (mean daily dose 852 mg) comparators in terms of the incidence of fatal and nonfatal heart attacks and strokes.

#### 3.3.1. Effect on Blood Pressure

The effects of NSAIDs on blood pressure (BP) have been shown to be more variable. The inhibition of prostaglandin synthesis by all NSAIDs can lead to impairment of renal function and a resulting increase in BP [61]. This is of particular importance for the elderly, who are more likely to have multiple comorbidities including concomitant hypertension and arthritis. Even a small reduction (2 mmHg decrease) in systolic BP (SBP) at a patient population level has been equated to clinically relevant reductions in CV risk (7% and 10% reduction in risk of mortality from ischemic heart disease and stroke, respectively) [62].

In comparison with BP measurements taken during the clinic visit, 24-hour ambulatory BP has recently been shown to be a better predictor of CV events [63] and may therefore be a more reliable measure for the assessment of CV safety.

There are relatively few studies of NSAIDs that use ambulatory BP measurements. One study in healthy older volunteers (aged 60–85 years) demonstrated that both etoricoxib (90 mg OD) and celecoxib (200 mg BID) on day 14 of treatment were associated with a significantly increased SBP compared with placebo (*p* ≤ 0.05), but the increase with etoricoxib (7.7 mmHg) was over three times greater than with celecoxib (2.4 mmHg) (*p* < 0.03) [61]. Two further ambulatory BP monitoring studies in patients with arthritis and concomitant CV risk factors or CV disease did not demonstrate mean changes with either celecoxib 200 mg OD over 6 weeks [64] or a standard dose of celecoxib 100 mg BID (with a mean daily dose of 208 mg) over 4 months [65]. The comparators in these studies were either associated with a similar neutral effect on mean SBP (naproxen 500 mg BID) [64] or a significant elevation in mean SBP of around 3.5 mmHg (rofecoxib 25 mg OD and ibuprofen 2031 mg mean daily dose) [64, 65]. The ambulatory BP effects of diclofenac have been investigated in relatively small populations (< 30 patients) [66, 67], and the data should therefore be treated cautiously. A consistent increase in mean SBP was seen in one crossover study of black and Hispanic patients when diclofenac (75 mg BID; 4.1 mmHg increase) was compared with celecoxib (200 mg OD; 0.6 mmHg increase) [66]. In a second, small crossover study of patients (aged 55–73 years) with diagnosed hypertension, a significant BP-raising effect was seen with only 75 mg/day of diclofenac, and intriguingly this effect was partially reversed by the use of the prostaglandin E2 analog, misoprostol, over the course of the 3-week study [67].

Meta-analyses, largely focused on investigator-reported changes in BP of the agents classified as coxibs, support the idea of some heterogeneity between agents. A meta-analysis of 114 trials comparing COX-2 selective inhibitors (rofecoxib, celecoxib, valdecoxib, parecoxib, etoricoxib, and lumiracoxib), conducted before June 2006, revealed significant heterogeneity in renal events (renal dysfunction, hypertension, and peripheral edema) between medicines in the class (*p* = 0.02), which was driven by rofecoxib [68]. The risks of both renal dysfunction and hypertension were lower with celecoxib compared with all controls [68]. The dataset considering etoricoxib was relatively small and only included one of the three trials in the MEDAL program. A later meta-analysis of 51 trials, considering all three component parts of the MEDAL program, confirmed the heterogeneity of the effect of different COX-2 selective inhibitors (celecoxib, rofecoxib, etoricoxib, valdecoxib, and lumiracoxib) on BP and hypertension [69], with etoricoxib (risk ratio [95% Cl], 1.52 [1.39–1.66],* p* < 0.01), but not celecoxib (0.89 [0.77–1.01],* p* = 0.22), being associated with increased risk of SBP elevation [69].

Looking specifically at the data from the MEDAL program, etoricoxib was associated with a higher overall incidence of hypertension-related AEs and discontinuations due to hypertension than diclofenac (Table 5) [35–37]. Additionally, in the MEDAL study alone, the mean (SD) change from baseline in SBP with etoricoxib (60 mg OA cohort) was also higher than with diclofenac (75 mg BID) (3.4 [0.19] and 1.6 [0.19], respectively) [70]. These differences have contributed to the additional product labeling for etoricoxib in some countries, to preclude the use of the medicine in hypertensive patients with persistently elevated BP above 140/90 mmHg and in whom adequate control has not been achieved [71]. No such contraindication is present in the prescribing information for celecoxib or diclofenac [72, 73]. The incidence of edema-related AEs was also significantly higher with etoricoxib than with diclofenac (Table 5) [36, 37]. The direct comparison between celecoxib and diclofenac is less robust, being based on a post hoc analysis of the CLASS trial—but there is no suggestion of a difference between medicines in the incidence of de novo hypertension, discontinuation due to hypertension, or edema (Table 5).

The reasons for differences in BP effects remain uncertain; however, a number of studies, both in vitro and in vivo, suggest that all three medicines considered here have the potential for both negative and in some cases positive effects on arterial function [50, 74–78]. The vast majority of these studies have focused on celecoxib and etoricoxib. Celecoxib was found to reduce oxidative stress and chronic inflammation and was also identified as a brachial artery vasodilator [74, 78], while etoricoxib appeared to have a neutral or negative effect on endothelial function in vivo [77]. At least some of these differences are believed to be due to the different physicochemical properties of the medicines (i.e., they are COX-independent), with their differing effects on plasma membrane phospholipids proposed to be important [77].

## 4. Conclusions

Data from various sources, including randomized controlled trials and meta-analyses, suggests that celecoxib, etoricoxib, and diclofenac are largely similar in their efficacy and CV safety, but celecoxib and etoricoxib are associated with less GI toxicity than diclofenac. Evidence for “hard” CV outcomes (heart attacks and strokes) does not allow for distinction between the three medicines; however, there may be molecule-specific properties that could explain why etoricoxib appears to be consistently associated with a more marked detrimental effect on BP.

## Figures and Tables

**Table 1:** Comparison of the efficacy of celecoxib and etoricoxib in patients with OA.

Study	Comparator drugs and doses	Difference between etoricoxib and celecoxib in TWA change from baseline over 12 weeks		
		WOMAC Pain Subscale (100 mm VAS)	WOMAC Physical Function Subscale (100 mm VAS)	PGADS (100 mm VAS)
Bingham III et al. 2007 [14]	Etoricoxib (30 mg OD), celecoxib (200 mg OD)	–3.12 (–7.02 to 0.77)^^a^^; *p *= 0.116	–1.74 (–5.53 to 2.05)^a^; *p *= 0.367	–4.05 (–8.11 to 0.02)^a^; *p *= 0.051

Bingham III et al. 2007 [14]	Etoricoxib (30 mg OD), celecoxib (200 mg OD)	0.14 (–3.72 to 4.00)^a^; *p *= 0.943	–0.08 (–3.83 to 3.67)^a^; *p *= 0.967	0.06 (–3.90 to 4.02)^a^; *p *= 0.977

Yoo et al. 2014 [15]	Etoricoxib (30 mg OD), celecoxib (200 mg OD)	–1.63 (–5.37 to 2.10)	–1.32 (–4.88 to 2.23)^^b^^	–1.09 (–5.84 to 3.30)^b^

Unless indicated otherwise, all efficacy endpoints are primary (or coprimary), and all data is shown as difference in LS mean change (95% CI).

^a^Etoricoxib is at least as effective as celecoxib when the upper bound of the 95% CI < 10 mm VAS. Negative mean difference value favors etoricoxib.

^b^Secondary endpoints.

OA, osteoarthritis; TWA, time-weighted average; WOMAC, Western Ontario and McMaster Universities Osteoarthritis Index; VAS, visual analog scale; PGADS, Patient Global Assessment of Disease Status; OD, once daily; LS, least squares.

**Table 2:** Summary of the double-blind randomized controlled studies comparing celecoxib or etoricoxib with diclofenac. Studies are arranged in chronological order from left to right.

			CLASS	EDGE	EDGE II	MEDAL study	MEDAL program	CONDOR
			Silverstein et al, 2000 [34]	Baraf, et al, 2007 [36]	Krueger et al, 2008 [37]	Combe et al, 2009 [38]	Cannon et al, 2006 [57]	Chan et al, 2010 [43]
NSAIDs	Celecoxib	Dose	400 mg BID^a^					200 mg BID
		*N*	3987					2238
	Diclofenac	Dose	75 mg BID	50 mg TID	75 mg BID	75 mg BID	50 mg TID OA or 75 mg BID OA or RA	75 mg BID
		*N*	1996	3518	2032	6700	17,289	2246
	Etoricoxib	Dose		90 mg OD	90 mg OD	60 mg OD OA or 90 mg OD OA or RA	60 mg OD OA or 90 mg OD OA or RA	
		*N*		3593	2054	6769^d^	17,412	

Aspirin		Allowed	YES	YES	YES	YES	YES	NO
		Dose	≤ 325 mg/day	< 100 mg/day	< 100 mg/day	< 100 mg/day	< 100 mg/day	
		% Patients	~21%^b^	~28%	~17%	~37%^d^	~35%	

PPIs		Allowed at investigator discretion	NO	YES	YES^c^	YES	YES	YES (part of treatment group)^e^

GI endpoints			(i) Upper GI bleeding (ii) GD perforation (iii) Obstruction (iv) Symptomatic GD ulcers	(i) Discontinuations due to clinical GI AEs (ii) Discontinuations due to laboratory GI AEs	(i) Discontinuations due to clinical GI AEs (ii) Discontinuations due to laboratory GI AEs	(i) Discontinuations due to clinical GI AEs (ii) Discontinuations due to laboratory GI AEs	(i) Upper GI bleeding (ii) GD perforation (iii) Obstruction (iv) Symptomatic GD ulcers	(i) Upper GI bleeding (ii) GD perforation (iii) Obstruction (iv) Bleeding (≥ 2 g/dL drop in Hb with visible lesion) (v) Bleeding (≥ 2 g/dL drop in Hb of presumed GI origin [adjudicated]) (vi) Visible lesions in lower GI tract

^a^Supratherapeutic dose of celecoxib (400 mg BID, double the maximum dose in RA and four times the maximum dose in OA).

^b^Data combined for diclofenac- and ibuprofen-treated patients (*N* = 1985; 800 mg TID).

^c^Recommended for patients at higher risk of upper GI clinical events (age > 65 years, history of ulcer or hemorrhage, concurrent use of corticosteroid, anticoagulant, or antiplatelet therapy).

^d^OA cohort, etoricoxib 60 mg.

^e^In patients taking diclofenac only.

NSAIDs, nonsteroidal anti-inflammatory drugs; BID, twice daily; TID, three times daily; OA, osteoarthritis; RA, rheumatoid arthritis; OD, once daily; PPIs, proton pump inhibitors; GI, gastrointestinal; GD, gastroduodenal; AEs, adverse events; Hb, hemoglobin.

**Table 3:** GI safety of celecoxib and etoricoxib compared with diclofenac in patients with OA and RA^a^.

Study	Reference	Celecoxib	Diclofenac	Etoricoxib
*Retrospective pooled analyses*				
Discontinuations due to any GI AEs, *n* (%)	NIculescu et al, 2009 [41]	613 (4.2)^c^	284 (5.0)*∗*	
Discontinuations due to GI intolerability AEs^b^, *n* (%)	NIculescu et al, 2009 [41]	510 (3.5)^c^	235 (4.2)*∗*	
Discontinuations due to GI AEs, *n* (%)	Mallen et al, 2011 [40]	283 (4.8)	115 (4.9)	
Discontinuations due to GI intolerability AEs^d^, *n* (%)	Mallen et al, 2011[40]	235 (4.0)	97 (4.2)	

*EDGE*				
Discontinuations due to GI AEs^e^, per 100 PY	Baraf et al, 2007 [36]		19.2	9.4
HR (95% Cl)			0.50 (0.43–0.58)*∗*	

*EDGE II*				
Discontinuations due to GI AEs^e^, per 100 PY	Krueger et al, 2008 [37]		8.5	5.2
HR (95% Cl)			0.62 (0.47–0.81)*∗*	

*MEDAL study (etoricoxib 60 mg OA cohort)*				
Discontinuations due to any clinical GI AEs^f^, *n* (%)	Combe et al, 2009 [38]		369 (5.5)*∗*	213 (3.1)
Discontinuations due to any laboratory GI/liver AEs, *n* (%)	Combe et al, 2009 [38]		96 (1.4)*∗*	9 (0.1)

*MEDAL program* ^g^				
Upper GI clinical events^f^, per 100 PY (95% CI)	Cannon et al, 2006 [57]		0.97 (0.85–1.10)	0.67 (0.57–0.77)
HR (95% Cl)			0.69 (0.57–0.83)	
Complicated upper GI clinical events^h^, per 100 PY	Lane et al, 2007 [39]		0.32	0.30
*n* (%)			82 (0.47)	78 (0.45)
HR (95% Cl)			0.91 (0.67–1.24)	
Uncomplicated upper GI clinical events^i^, per 100 PY	Lane et al, 2007 [39]		0.65	0.37
*n* (%)			164 (0.95)	98 (0.56)
HR (95% Cl)			0.57 (0.45–0.74)	
Lower GI clinical events^j^, per 100 PY (95% Cl)	Lane et al, 2008 [45]		0.38 (0.31–0.46)	0.32 (0.25–0.39)
HR (95% Cl)			0.84 (0.63–1.13)	

*CONDOR*				
Clinically significant events throughout the GI tract^k^, *n* (%)	Chan et al, 2010 [43]	20 (0.9)	81 (3.8)*∗*	
HR (95% Cl)		4.3 (2.6–7.0)		
Discontinued due to GI AEs, n (%)	Chan et al, 2010 [43]	114 (6.0)	167 (8.0)*∗*	

^a^In addition to GI safety, data from parts of the MEDAL clinical trial programs and the CLASS study demonstrated a significantly higher incidence of hepatic AEs with diclofenac than with either coxib comparator (celecoxib versus diclofenac: 0.6% and 2.3%, respectively, *p *≤ 0.05; etoricoxib versus diclofenac: 4.3% and 9.4%, respectively, *p *≤ 0.05) (Silverstein et al. 2000, Krueger et al. 2008).

^b^GI intolerability AEs: abdominal pain, diarrhea, dyspepsia, flatulence, and nausea.

^c^Celecoxib combined doses of 200 mg daily dose and 400 mg daily dose.

^d^GI intolerability AEs: abdominal pain, constipation, diarrhea, dyspepsia, flatulence, and nausea.

^e^Clinical and laboratory GI AEs.

^f^Bleeding from the esophagus, stomach, or duodenum; perforation or obstruction from a nonmalignant gastric or duodenal ulcer; or an ulcer documented on clinically indicated workup.

^g^The MEDAL program consists of data from the MEDAL, EDGE I, and EDGE II trials.

^h^Perforation, obstruction, and witnessed ulcer or significant bleeding.

^i^Uncomplicated ulcer or bleeding.

^j^Bleeding, perforation, obstruction.

^k^Gastroduodenal, small-bowel, or large-bowel hemorrhage, gastric-outlet obstruction; gastroduodenal, small-bowel, or large-bowel perforation; clinically significant anemia of defined GI or presumed occult GI origin; acute GI hemorrhage of unknown origin.

*∗p* ≤ 0.05.

GI, gastrointestinal; OA, osteoarthritis; RA, rheumatoid arthritis; AEs, adverse events; PY, patient-years; HR, hazard ratio.

**Table 4:** CV safety of celecoxib, etoricoxib, and diclofenac in patients with OA and RA.

	Meta-analyses				
Endpoints	Reference	Comparator	Celecoxib	Diclofenac	Etoricoxib
APTC composite outcome^a^, rate ratio (95% CI)	Trelle et al, 2011 [54]	Placebo	1.43 (0.94–2.16)	1.60 (0.85–2.99)	1.53 (0.74–3.17)
APTC-like composite outcome^b^, RR (95% CI)	Bhala et al, 2013 [11]	Diclofenac	0.94 (0.54–1.63)		1.01 (0.87–1.18)

	Randomized controlled trials				
Endpoints	Reference	Celecoxib	Diclofenac		Etoricoxib

Thrombotic events, rate (95% Cl)^c^	Cannon et al, 2006 [57]		1.30 (1.17–1.45)		1.24 (1.11–1.38)
APTC^d^ events, rate (95% Cl)^c^	Cannon et al, 2006 [57]		0.87 (0.76–1.00)		0.84 (0.73–0.95)
Serious CV thromboembolic events^e^, %	White et al, 2002 [59]	1.3	1.4		

^a^Myocardial infarction, stroke, and vascular death.

^b^Nonfatal myocardial infarction, nonfatal stroke, and vascular death.

^c^Per 100 patient-years at risk, per-protocol comparison.

^d^Myocardial infarction, stroke, and vascular death.

^e^Including cardiac, cerebrovascular, and peripheral vascular events.

*∗p *< 0.0033.

OA, osteoarthritis; RA, rheumatoid arthritis; APTC, Antiplatelet Trialists' Collaboration; RR, relative ratio; RRR, pooled ratio of relative risks; CV, cardiovascular.

**Table 5:** Renal safety of celecoxib and etoricoxib compared with diclofenac in patients with OA and RA in randomized controlled trials.

	Reference	Study duration	Celecoxib	Diclofenac	Etoricoxib
*Hypertension *					
Hypertension^a,b^, *n* (%)	White et al, 2002 [59]	Minimum 6 months	109 (2.7)	52 (2.6)	
Discontinued due to any hypertension-related AEs, %	Whelton et al, 2006 [79]	Minimum 6 months	0.3	0.2	
Incidence of hypertension-related AEs, *n* (%)	Krueger et al, 2008 [37]	Mean ~19 months		313 (15.2)*∗*	397 (19.5)
Discontinued due to any hypertension-related AEs, *n* (%)	Baraf et al, 2007 [36]	Mean ~9 months		23 (0.7)*∗*	81 (2.3)

*Edema*					
Edema-related AEs, %	Whelton et al, 2006 [79]	Minimum 6 months	4.1	4.1	
Incidence of edema-related AEs, *n* (%)	Krueger et al, 2008 [37]	Mean ~19 months		94 (4.6)*∗*	132 (6.5)

*Renal*					
Discontinuations due to renal dysfunction^c^, %	Cannon et al, 2006 [57]	Mean ~18 months		0.8	0.8

^a^New-onset and aggravated pre-existing.

^b^For ibuprofen (800 mg TID) 4.2%, *p *< 0.05 versus celecoxib.

^c^OA cohort etoricoxib 60 mg.

*∗p *≤ 0.05.

OA, osteoarthritis; RA, rheumatoid arthritis; AEs, adverse events.

## References

[1] Mitchell J. A., Akarasereenont P., Thiemermann C., Flower R. J., Vane J. R. (1993). Selectivity of nonsteroidal antiinflammatory drugs as inhibitors of constitutive and inducible cyclooxygenase. *Proceedings of the National Acadamy of Sciences of the United States of America*.

[2] Vane J. R., Bakhle Y. S., Botting R. M. (1998). Cyclooxygenases 1 and 2. *Annual Review of Pharmacology and Toxicology*.

[3] Schwartz J. I., Dallob A. L., Larson P. J. (2008). Comparative inhibitory activity of etoricoxib, celecoxib, and diclofenac on COX-2 versus COX-1 in healthy subjects. *Clinical Pharmacology and Therapeutics*.

[4] Hunter T. S., Robison C., Gerbino P. P. (2015). Emerging evidence in NSAID pharmacology: important considerations for product selection. *The American Journal of Managed Care*.

[5] Schmidt M., Lamberts M., Olsen A. S. (2016). Cardiovascular safety of non-aspirin non-steroidal anti-inflammatory drugs: review and position paper by the working group for Cardiovascular Pharmacotherapy of the European Society of Cardiology. *European Heart Journal - Cardiovascular Pharmacotherapy*.

[6] Gierse J. K., Koboldt C. M., Walker M. C., Seibert K., Isakson P. C. (1999). Kinetic basis for selective inhibition of cyclo-oxygenases. *Biochemical Journal*.

[7] Warner T. D., Giuliano F., Vojnovic I., Bukasa A., Mitchell J. A., Vane J. R. (1999). Nonsteroid drug selectivities for cyclo-oxygenase-1 rather than cyclo-oxygenase-2 are associated with human gastrointestinal toxicity: a full *in vitro* analysis. *Proceedings of the National Acadamy of Sciences of the United States of America*.

[8] Patrono C., Patrignani P., García Rodríguez L. A. (2001). Cyclooxygenase-selective inhibition of prostanoid formation: transducing biochemical selectivity into clinical read-outs. *The Journal of Clinical Investigation*.

[9] Tacconelli S., Capone M. L., Sciulli M. G., Ricciotti E., Patrignani P. (2002). The biochemical selectivity of novel COX-2 inhibitors in whole blood assays of COX-isozyme activity. *Current Medical Research and Opinion*.

[10] Capone M. L., Tacconelli S., Di Francesco L., Sacchetti A., Sciulli M. G., Patrignani P. (2007). Pharmacodynamic of cyclooxygenase inhibitors in humans. *Prostaglandins & Other Lipid Mediators*.

[11] Bhala N., Emberson J., Merhi A. (2013). Vascular and upper gastrointestinal effects of non-steroidal anti-inflammatory drugs: meta-analyses of individual participant data from randomised trials. *The Lancet*.

[12] European Medicines Agency Diclofenac-containing medicines. http://www.ema.europa.eu/ema/index.jsp?curl=pages/medicines/human/referrals/Diclofenac-containing_medicines/human_referral_prac_000009.jsp&mid=WC0b01ac05805c516f.

[13] Medicines and Healthcare products Regulatory Agency Diclofenac tablets now only available as a prescription medicine. https://www.gov.uk/government/news/diclofenac-tablets-now-only-available-as-a-prescription-medicine.

[14] Bingham C. O., Sebba A. I., Rubin B. R. (2007). Efficacy and safety of etoricoxib 30 mg and celecoxib 200 mg in the treatment of osteoarthritis in two identically designed, randomized, placebo-controlled, non-inferiority studies. *Rheumatology*.

[15] Yoo M. C., Yoo W. H., Kang S. B. (2014). Etoricoxib in the treatment of Korean patients with osteoarthritis in a double-blind, randomized controlled trial. *Current Medical Research and Opinion*.

[16] McKenna F., Borenstein D., Wendt H., Wallemark C., Lefkowith J. B., Geis G. S. (2001). Celecoxib versus diclofenac in the management of osteoarthritis of the knee: A placebo-controlled, randomised, double-blind comparison. *Scandinavian Journal of Rheumatology*.

[17] Zacher J., Feldman D., Gerli R. (2003). A comparison of the therapeutic efficacy and tolerability of etoricoxib and diclofenac in patients with osteoarthritis. *Current Medical Research and Opinion*.

[18] Gottesdiener K., Schnitzer T., Fisher C. (2002). Results of a randomized, dose-ranging trial of etoricoxib in patients with osteoarthritis. *Rheumatology*.

[19] Emery P., Koncz T., Pan S., Lowry S. (2008). Analgesic effectiveness of celecoxib and diclofenac in patients with osteoarthritis of the hip requiring joint replacement surgery: a 12-week, multicenter, randomized, double-blind, parallel-group, double-dummy, noninferiority study. *Clinical Therapeutics*.

[20] Emery P., Zeidler H., Kvien T. K. (1999). Celecoxib versus diclofenac in long-term management of rheumatoid arthritis: Randomised double-blind comparison. *The Lancet*.

[21] Sieper J., Klopsch T., Richter M. (2008). Comparison of two different dosages of celecoxib with diclofenac for the treatment of active ankylosing spondylitis: Results of a 12-week randomised, double-blind, controlled study. *Annals of the Rheumatic Diseases*.

[22] Huang F., Gu J., Liu Y. (2014). Efficacy and Safety of Celecoxib in Chinese Patients with Ankylosing Spondylitis: A 6-Week Randomized, Double-Blinded Study with 6-Week Open-Label Extension Treatment. *Current Therapeutic Research*.

[23] Walker C., Essex M. N., Li C., Park P. W. (2016). Celecoxib versus diclofenac for the treatment of ankylosing spondylitis: 12-week randomized study in Norwegian patients. *Journal of International Medical Research*.

[24] Sweeney S., Taylor G., Calin A. (2002). The effect of a home based exercise intervention package on outcome in ankylosing spondylitis: a randomized controlled trial. *The Journal of Rheumatology*.

[25] Van Der Heijde D., Baraf H. S. B., Ramos-Remus C. (2005). Evaluation of the efficacy of etoricoxib in ankylosing spondylitis: Results of a fifty-two-week, randomized, controlled study. *Arthritis & Rheumatology*.

[26] Sieper J., Listing J., Poddubnyy D. (2016). Effect of continuous versus on-demand treatment of ankylosing spondylitis with diclofenac over 2 years on radiographic progression of the spine: Results from a randomised multicentre trial (ENRADAS). *Annals of the Rheumatic Diseases*.

[27] Wanders A., Van Der Heijde D., Landewé R. (2005). Nonsteroidal antiinflammatory drugs reduce radiographic progression in patients with ankylosing spondylitis: A randomized clinical trial. *Arthritis & Rheumatology*.

[28] Zhu J., Sounthonevat C., Walker C. (2017). Celecoxib for the Treatment of Ankylosing Spondylitis. *Journal of Rheumatology and Arthritic Diseases*.

[29] Zweers M. C., de Boer T. N., van Roon J., Bijlsma J. W., Lafeber F. P., Mastbergen S. C. (2011). Celecoxib: considerations regarding its potential disease-modifying properties in osteoarthritis. *Arthritis Research and Therapy*.

[30] Jendrossek V. (2013). Targeting apoptosis pathways by Celecoxib in cancer. *Cancer Letters*.

[31] Moon Y. (2017). NSAID-activated gene 1 and its implications for mucosal integrity and intervention beyond NSAIDs. *Pharmacological Research*.

[32] Bjarnason I., Scarpignato C., Holmgren E., Olszewski M., Rainsford K. D., Lanas A. (2018). Mechanisms of Damage to the Gastrointestinal Tract From Nonsteroidal Anti-Inflammatory Drugs. *Gastroenterology*.

[33] Wallace J. L. (2008). Prostaglandins, NSAIDs, and gastric mucosal protection: why doesn't the stomach digest itself?. *Physiological Reviews*.

[34] Silverstein F. E., Faich G., Goldstein J. L. (2000). Gastrointestinal toxicity with celecoxib vs nonsteroidal anti-inflammatory drugs for osteoarthritis and rheumatoid arthritis: the CLASS study: a randomized controlled trial. *The Journal of the American Medical Association*.

[35] Cannon C. P., Curtis S. P., FitzGerald G. A. (2006). Cardiovascular outcomes with etoricoxib and diclofenac in patients with osteoarthritis and rheumatoid arthritis in the Multinational Etoricoxib and Diclofenac Arthritis Long-term (MEDAL) programme: a randomised comparison. *The Lancet*.

[36] Baraf H. S. B., Fuentealba C., Greenwald M. (2007). Gastrointestinal side effects of etoricoxib in patients with osteoarthritis: results of the etoricoxib versus diclofenac sodium gastrointestinal tolerability and effectiveness (EDGE) trial. *The Journal of Rheumatology*.

[37] Krueger K., Lino L., Dore R. (2008). Gastrointestinal tolerability of etoricoxib in rheumatoid arthritis patients: results of the etoricoxib vs diclofenac sodium gastrointestinal tolerability and effectiveness trial (EDGE-II). *Annals of the Rheumatic Diseases*.

[38] Combe B., Swergold G., McLay J. (2009). Cardiovascular safety and gastrointestinal tolerability of etoricoxib vs diclofenac in a randomized controlled clinical trial (The MEDAL study). *Rheumatology*.

[39] Laine L., Curtis S. P., Cryer B., Kaur A., Cannon C. P. (2007). Assessment of upper gastrointestinal safety of etoricoxib and diclofenac in patients with osteoarthritis and rheumatoid arthritis in the Multinational Etoricoxib and Diclofenac Arthritis Long-term (MEDAL) programme: a randomised comparison. *The Lancet*.

[40] Mallen S. R., Essex M. N., Zhang R. (2011). Gastrointestinal tolerability of NSAIDs in elderly patients: A pooled analysis of 21 randomized clinical trials with celecoxib and nonselective NSAIDs. *Current Medical Research and Opinion*.

[41] Niculescu L., Li C., Huang J., Mallen S. (2009). Pooled analysis of GI tolerability of 21 randomized controlled trials of celecoxib and nonselective NSAIDs. *Current Medical Research and Opinion*.

[42] Wallace J. L. (2012). NSAID gastropathy and enteropathy: Distinct pathogenesis likely necessitates distinct prevention strategies. *British Journal of Pharmacology*.

[43] Chan F. K. L., Lanas A., Scheiman J., Berger M. F., Nguyen H., Goldstein J. L. (2010). Celecoxib versus omeprazole and diclofenac in patients with osteoarthritis and rheumatoid arthritis (CONDOR): A randomised trial. *The Lancet*.

[44] Chan F. K. L., Hung L. C. T., Suen B. Y. (2002). Celecoxib versus diclofenac and omeprazole in reducing the risk of recurrent ulcer bleeding in patients with arthritis. *The New England Journal of Medicine*.

[45] Laine L., Curtis S. P., Langman M. (2008). Lower Gastrointestinal Events in a Double-Blind Trial of the Cyclo-Oxygenase-2 Selective Inhibitor Etoricoxib and the Traditional Nonsteroidal Anti-Inflammatory Drug Diclofenac. *Gastroenterology*.

[46] Fornai M., Antonioli L., Colucci R. (2014). NSAID-induced enteropathy: are the currently available selective COX-2 inhibitors all the same?. *The Journal of Pharmacology and Experimental Therapeutics*.

[47] Bjarnason I., Takeuchi K. (2009). Intestinal permeability in the pathogenesis of NSAID-induced enteropathy. *Journal of Gastroenterology*.

[48] Gwee K. A., Goh V., Lima G., Setia S. (2018). Coprescribing proton-pump inhibitors with nonsteroidal anti-inflammatory drugs: Risks versus benefits. *Journal of Pain Research*.

[49] McAdam B. F., Catella-Lawson F., Mardini I. A., Kapoor S., Lawson J. A., FitzGerald G. A. (1999). Systemic biosynthesis of prostacyclin by cyclooxygenase (COX)-2: The human pharmacology of a selective inhibitor of COX-2. *Proceedings of the National Acadamy of Sciences of the United States of America*.

[50] Mukherjee D., Nissen S. E., Topol E. J. (2001). Risk of cardiovascular events associated with selective COX-2 inhibitors. *The Journal of the American Medical Association*.

[51] Bresalier R. S., Sandler R. S., Quan H. (2005). Cardiovascular events associated with rofecoxib in a colorectal adenoma chemoprevention trial. *The New England Journal of Medicine*.

[52] Bertagnolli M. M., Eagle C. J., Zauber A. G. (2006). Celecoxib for the prevention of sporadic colorectal adenomas. *The New England Journal of Medicine*.

[53] Arber N., Eagle C. J., Spicak J. (2006). Celecoxib for the prevention of colorectal adenomatous polyps. *The New England Journal of Medicine*.

[54] Trelle S., Reichenbach S., Wandel S. (2011). Cardiovascular safety of non-steroidal anti-inflammatory drugs: network meta-analysis. *British Medical Journal*.

[55] McGettigan P., Henry D. (2011). Cardiovascular risk with non-steroidal anti-inflammatory drugs: systematic review of population-based controlled observational studies. *PLoS Medicine*.

[56] Martín Arias L. H., Martín González A., Sanz Fadrique R., Vazquez E. S. (2018). Cardiovascular Risk of Nonsteroidal Anti-inflammatory Drugs and Classical and Selective Cyclooxygenase-2 Inhibitors: A Meta-analysis of Observational Studies. *The Journal of Clinical Pharmacology*.

[57] Cannon C. P., Curtis S. P., Bolognese J. A., Laine L. (2006). Clinical trial design and patient demographics of the Multinational Etoricoxib and Diclofenac Arthritis Long-term (MEDAL) Study Program: Cardiovascular outcomes with etoricoxib versus diclofenac in patients with osteoarthritis and rheumatoid arthritis. *American Heart Journal*.

[58] Medicines and Healthcare products Regulatory Agency Etoricoxib (Arcoxia): revised dose recommendation for rheumatoid arthritis and ankylosing spondylitis. https://www.gov.uk/drug-safety-update/etoricoxib-arcoxia-revised-dose-recommendation-for-rheumatoid-arthritis-and-ankylosing-spondylitis.

[59] White W. B., Faich G., Whelton A. (2002). Comparison of thromboembolic events in patients treated with celecoxib, a cyclooxygenase-2 specific inhibitor, versus ibuprofen or diclofenac. *American Journal of Cardiology*.

[60] Nissen S. E., Yeomans N. D., Solomon D. H. (2016). Cardiovascular safety of celecoxib, naproxen, or ibuprofen for arthritis. *The New England Journal of Medicine*.

[61] Schwartz J. I., Thach C., Lasseter K. C. (2007). Effects of etoricoxib and comparator nonsteroidal anti-inflammatory drugs on urinary sodium excretion, blood pressure, and other renal function indicators in elderly subjects consuming a controlled sodium diet. *Clinical Pharmacology and Therapeutics*.

[62] Lewington S., Clarke R., Qizilbash N., Peto R., Collins R. (2002). Age-specific relevance of usual blood pressure to vascular mortality: a meta-analysis of individual data for one million adults in 61 prospective studies. *The Lancet*.

[63] Banegas J. R., Ruilope L. M., de la Sierra A. (2018). Relationship between Clinic and Ambulatory Blood-Pressure Measurements and Mortality. *The New England Journal of Medicine*.

[64] Sowers J. R., White W. B., Pitt B. (2005). The effects of cyclooxygenase-2 inhibitors and nonsteroidal anti-inflammatory therapy on 24-hour blood pressure in patients with hypertension, osteoarthritis, and type 2 diabetes mellitus. *Archives of Internal Medicine*.

[65] Ruschitzka F., Borer J. S., Krum H. (2017). Differential blood pressure effects of ibuprofen, naproxen, and celecoxib in patients with arthritis: the PRECISION-ABPM (Prospective Randomized Evaluation of Celecoxib Integrated Safety Versus Ibuprofen or Naproxen Ambulatory Blood Pressure Measurement) Trial. *European Heart Journal*.

[66] Izhar M., Alausa T., Folker A., Hung E., Bakris G. L. (2004). Effects of COX Inhibition on Blood Pressure and Kidney Function in ACE Inhibitor-Treated Blacks and Hispanics. *Hypertension*.

[67] Munger M. A., Gardner S. F., Ateshkadi A. (2008). Misoprostol effects on diclofenac-induced cardiorenal changes in salt-sensitive patients with hypertension: The MEDIC study. *Pharmacotherapy*.

[68] Zhang J., Ding E. L., Song Y. (2006). Adverse effects of cyclooxygenase 2 inhibitors on renal and arrhythmia events: meta-analysis of randomized trials. *The Journal of the American Medical Association*.

[69] Chan C. C., Reid C. M., Aw T.-J., Liew D., Haas S. J., Krum H. (2009). Do COX-2 inhibitors raise blood pressure more than nonselective NSAIDs and placebo? An updated meta-analysis. *Journal of Hypertension*.

[70] Krum H., Swergold G., Curtis S. P. (2009). Factors associated with blood pressure changes in patients receiving diclofenac or etoricoxib: results from the MEDAL study. *Journal of Hypertension*.

[71] Merck Sharp & Dohme Ltd Arcoxia 30 60 90 120 mg film-coated tablets [prescribing information]. https://www.medicines.org.uk/emc/product/3302/smpc.

[72] Accord-UK Ltd Diclofenac potassium 50 mg tablets prescribing information. https://www.medicines.org.uk/emc/product/4515/smpc.

[73] Pfizer Ltd Celebrex 100mg capsule [prescribing information]. https://www.medicines.org.uk/emc/product/5533/smpc.

[74] Chenevard R., Hürlimann D., Béchir M. (2003). Selective COX-2 inhibition improves endothelial function in coronary artery disease. *Circulation*.

[75] Crilly M. A., Mangoni A. A., Knights K. M. (2013). Aldosterone glucuronidation inhibition as a potential mechanism for arterial dysfunction associated with chronic celecoxib and diclofenac use in patients with rheumatoid arthritis. *Clinical and Experimental Rheumatology*.

[76] Hinz B., Dormann H., Brune K. (2006). More pronounced inhibition of cyclooxygenase 2, increase in blood pressure, and reduction of heart rate by treatment with diclofenac compared with celecoxib and rofecoxib. *Arthritis & Rheumatology*.

[77] Walter M. F., Jacob R. F., Day C. A., Dahlborg R., Weng Y., Mason R. P. (2004). Sulfone COX-2 inhibitors increase susceptibility of human LDL and plasma to oxidative modification: Comparison to sulfonamide COX-2 inhibitors and NSAIDs. *Atherosclerosis*.

[78] Widlansky M. E., Price D. T., Gokce N. (2003). Short- and long-term COX-2 inhibition reverses endothelial dysfunction in patients with hypertension. *Hypertension*.

[79] Whelton A., Lefkowith J. L., West C. R., Verburg K. M. (2006). Cardiorenal effects of celecoxib as compared with the nonsteroidal anti-inflammatory drugs diclofenac and ibuprofen. *Kidney International*.

