# Erratum to “Are All Oral COX-2 Selective Inhibitors the Same? A Consideration of Celecoxib, Etoricoxib, and Diclofenac”

**DOI:** 10.1155/2019/8635073

**Published:** 2019-06-02

**Authors:** Chris Walker

**Affiliations:** Pfizer, Walton Oaks, UK

In the article titled “Are All Oral COX-2 Selective Inhibitors the Same? A Consideration of Celecoxib, Etoricoxib, and Diclofenac” [1], there was an error in reference [24] where it should be replaced with the article titled “A randomized, controlled, clinical trial of etoricoxib in the treatment of rheumatoid arthritis” [2].
